# What do outlet’s and provider’s characteristics mean for family planning consumers? A comparative study of Kenya, Nigeria and Uganda

**DOI:** 10.1186/s12905-023-02699-0

**Published:** 2023-10-16

**Authors:** Truc Ngoc Hoang Dang, Duc Dung Le, Sutthida Chuanwan, Duah Dwomoh

**Affiliations:** 1 Institute for Population and Social Research, Mahidol University, Nakhon Pathom, Thailand; 2 Institute of Social and Medical Studies, Hanoi, Vietnam; 3 Department of Biostatistics, University of Ghana, Accra, Ghana

**Keywords:** Family planning, Consumer market for family planning, Kenya, Nigeria, Uganda

## Abstract

**Background:**

This research investigated the determinants of the number of family planning consumers in Kenya, Nigeria and Uganda, with a focus on outlet’s and provider’s characteristics which are important factors influencing the choice of using contraceptive methods but largely unexplored in previous literature.

**Methods:**

We utilized a unique panel survey on outlet’s in Kenya (n = 1,321), Nigeria (n = 1,255) and Uganda (n = 842), which is part of the Consumer Market for Family Planning conducted in between 2019 and 2020, for the analysis of the pooled data (n = 3,418) and individual country. Random effects Poisson regressions were performed.

**Results:**

The pooled data results showed that the expected number of consumers were significantly lower in Nigeria and Uganda than in Kenya, and that working experience (provider’s characteristics), types of stores, duration of providing family planning services, participations in community outreach and host community events, and sign of family planning services (outlet’s characteristics) were significant determinants of the number of customers. The results for each country revealed interesting similarities and differences in the determinants across the three countries.

**Conclusions:**

This study sheds light on the relationship between the number of family planning customers and outlet’s and provider’s characteristics, thus providing informative evidence-based to on-going debates on the coverage of family planning services, which is still insufficient in developing countries. As a result, the government’s family planning expenditures should instead prioritize small, private enterprises such as pharmacies or drug stores. Furthermore, it is critical to focus on several critical tasks to improve the qualities of outlets and providers to attract customers, such as ensuring that they are eye-catching, advertising FP services, have professional credentials, fulfil providers’ obligations to counsel contraceptive users, provide long-term services and community care, and have female providers.

**Supplementary Information:**

The online version contains supplementary material available at 10.1186/s12905-023-02699-0.

## Introduction

Family planning (FP), one of the target goals of the 2030 Sustainable Development Goals (SDGs), has been considered an emerging global public health issue in low-income countries, especially in Sub-Saharan African countries where the total fertility rates are still high. In particular, ensuring universal access to sexual and reproductive health care services including FP and the integration of reproductive health into national programs and strategies, are adopted in goal 3.7 of SDGs [1]. Contraceptive use and reducing unmet need are the key methods in FP programs [1, 2]. In response to this issue, a global partnership of FP stakeholders including governments, private sectors and civil society organizations has been established aiming to raise the number of additional modern contraceptive users in developing regions to 120 million by 2020 [3]. Corresponding new goals for the partnership in 2030 are in progress, with the aim of ensuring that all individuals can make their own informed decisions about the use of contraception [4]. In addition, leading international organizations have shown their strong efforts and commitments in increasing access to sexual and reproductive health services, including FP [1]. Although impressive progress has been made, the goal of achieving 120 million users has not been successful [3].

Previous studies on FP consumers or contraceptive use in Kenya, Nigeria and Uganda have focused on two main directions. The first direction, the prominent research strand, mostly uses Demographic Health Survey (DHS) to identify the determinants of contraceptive methods among individuals, especially among women of reproductive age (WRA) (e.g., 15–49 years old). Specifically, studies in Kenya found that age, marital status, the number of children, education, household wealth, geographic areas, and partner’s attitude are associated with women’s choices of contraceptive methods [5–7]. Similarly, previous literature in Uganda proved that age, age at first birth, education, household wealth, and geographic areas are significant predictors of contraceptive use [8–10]. Similar socio-demographic factors, religion and healthcare access also have been found to be determinants of contraceptive uptake among Nigerian women and couples [11–13].

The second direction, the narrower research strand, investigates the determinants of client satisfaction or unmet need and demands for FP [14–16]. While these studies have provided informative evidence on the determinants of contraceptive services across the three countries under examination, none of them have focused on the supply side of FP services. Generally, the demand for contraceptive methods is influenced by knowledge, awareness and attitudes of users. When there is a demand, it calls for a supply. The supply side of FP services refers to characteristics of outlets/clinics (e.g., types, location, opening hours and availability of FP methods) and providers (e.g., socio-demographic, well-equipped and trained staff). Previous studies have suggested that the provision of FP resources and health facilities [17, 18] have played important roles in influencing the use of FP methods, and that failing to provide appropriate FP supply services might affect reproductive health issues among women such as unintended pregnancies and abortions [19, 20]. Furthermore, lack of supply services has been arguably responsible for the failure of FP programs in some developing countries [18, 21].

To better understand the determinants of the number of FP users, it is important to account for outlets’ and providers’ characteristics because it may affect clients’ satisfaction and their decision on where to use the services. In an attempt in bridging the research gap, this study used three-panel surveys on the Consumer’s Market for Family Planning (CM4FP) project to explore the relationship between the number of clients and outlet’ and provider’s characteristics across these three African countries with different income levels: Nigeria being the richest, Uganda being the poorest, and Kenya falling into the lower-middle-income category based on income classifications by the World Bank in the year 2021 and 2022 [22]. the CM4FP), surveyed from 2019 to 2020, intended to gain a better knowledge of the supply and demand sides of the FP market in Kenya, Uganda, and Nigeria and thus is well-suited for this study.

## Country context

In Africa, although the number of people seeking modern methods of FP, such as the pill (oral contraceptives), intrauterine device (IUD), injectables, female sterilization, male sterilization, female condoms, male condoms, implants, emergency contraception, among other methods climbed from 55 to 58% between 2015 and 2020, it was still lower than the global average, which was at 77% in 2020 [1, 23]. According to the United Nations, the proportion of women in Sub-Saharan Africa satisfying with modern contraception remains low [1]. Kenya, Nigeria, and Uganda are among the African countries with high fertility rates [24]. In the previous 70 years, the average total fertility rate (TFR) – defined as the average number of children a woman would have during her reproductive years (typically ages 15 to 49) – has been six and seven children per woman in the three countries under examination [24]. According to the United Nations, Uganda and Nigeria’s TFR decreased somewhat from seven children in 1950 to five children in 2015–2020 [25]. Kenya’s TFR dropped to 3.5 children in the same time span, owing to economic progress and the effectiveness of the national FP program.

Kenya, Nigeria and Uganda have committed to form a FP 2030 cooperation to advance rights-based FP in order to maintain a reduction in fertility [26]. Several studies have established that Kenya has made significant successes in increasing access to FP, particularly among married women [27–29]. According to estimates, about 44% of Kenyan women aged 15 to 49 used contraceptives in 2021, either in the form of traditional or modern contraceptive methods. The FP prevalence use rates were relatively high, reaching over 60% among married women or in a partnership, and it was found higher in metropolitan areas [30, 31].

The Nigerian government has set a target of achieving a 27% contraceptive prevalence rate by 2024, focusing on debunking myths and misconceptions regarding FP [26, 32]. According to National Bureau of Statistics [32], the contraceptive prevalence rate in Nigeria was at 17% among all women and at 20% among married Nigerian women or those in a union.

Uganda also has a low rate of contraception use. The Ugandan government has pledged to implement the Family Planning 2030 strategy aiming to reduce unmet need for FP among teenagers from 30.4% to 2016 to 25% in 2021, as well as enhancing service delivery with mixed methods. It has been found that married women or women in a partnerships utilize contraception at a higher rate than unmarried women (30% for unmarried women and 39% among married women) [26]. In conclusion, the similarities and differences in the country contexts in FP issues and contraceptive prevalence rates of the three countries are well-fitted and thus providing a good opportunity to conduct this study.

## Methods

### Study population and data source

This study used datasets from the CM4FP project consisting of a multi-round longitudinal FP outlet census and a cross-sectional household survey on history, experiences and perceptions of accessing FP services among women aged 18–49. Data was collected quarterly in each country, from 2019 to 2020 [33]. The goal of the project was to examine the feasibility and utility of various novel methodological approaches to have a deeper understanding of the supply and demand sides of the FP market in Kenya, Nigeria and Uganda. By adopting novel approaches, FP users can be directly linked to the outlets where the most recent FP methods of users have been recorded, and thus the project also wishes to overcome the constraints of existing FP data sources, which preclude a complete knowledge of the FP market. The project was implemented by Population Services International (PSI) in collaboration with Population Services Kenya, Society for Family Health Nigeria and PSI Uganda [33].

Regarding the sampling method of the outlet census^1^, the project initially chose 12 sites (four for each country) in an urban area with different sizes in geography, ranging from small to large areas. The site selection was based on various elements such as donor preference and PSI programmatic interest. Within each site, the project employed a ring-fenced approach to enumerate and map all outlets falling with the ring. Initially, a target of 600 outlets across all sites in each country was set. Fieldwork was implemented to survey all outlets across sectors and levels if the outlets provided FP methods/services of all kinds, except those offering only male condoms. Specifically, the outlet survey included a background information about outlet characteristics such as types of outlets, sector, service availability, current stock including brand name, formulations and price offered to consumers, current and recent stock-outs, client volume by method types, client fees, and sources of FP commodities and local distribution activities. The targeted outlets were pharmacies, drug shops, medical centres, clinics, health centers and hospitals. The fieldwork continued until the desired sample size in each country was obtained. Data were collected on a quarterly basis in a nine-month period of the project, starting 2019 and ending in 2020. In sum, the project had successfully implemented three rounds in Kenya and Nigeria. Due to late inclusion, two rounds of data collection were completed in Uganda [34, 35].

In Kenya, the baseline survey consisted of 603 outlets, of which 558 outlets (response rates: 92.5%) and 495 (response rates: 82.1%) were followed in the second and third rounds. To make up for the number of outlets lost due to the attrition, 52 and 9 new outlets were added in rounds 2 and 3, respectively. In total, we had 1,753 observations (664 unique outlets) in Kenya. Similarly, 647 outlets were surveyed at the baseline in Nigeria and among them 619 (response rates: 96.7%) and 481 (response rates: 74.3%) were re-interviewed in rounds 2 and 3. These two rounds also surveyed 39 new outlets (16 in round 2 and 23 in round 3). Thus, in Nigeria, we had 1,786 observations (672 unique outlets). As for Uganda, there were 485 outlets at the baseline. There were 63 (response rates: 87%) outlets were dropped out and 15 newly added in the second round. Thus, we had 922 observations (500 unique outlets). After dropping missing values in variables of interest for the analysis, we had 1,321 observations for Kenya, 1,255 observations for Nigeria, 842 observations for Uganda. For pooled dataset of the three countries, we had 3,418 observations.

### Measures

*Dependent variable*: Total number of family planning clients reported by the facilities^2^ per week (a count variable).

*Independent variables*: we used two groups of independent variables including outlet’s and provider’s characteristics^3^. The descriptions of these characteristics are as follows:

*Provider’s characteristics*: covered a set of questions about sex, age in years, levels of education, working experience at the outlet in years, FP training participation in the last 12 months.

*Outlet’s characteristics*: covered a group of characteristics of the outlet, such as types of outlets, duration of providing FP services or products, participation in community outreach and host FP community outreach events, availability of a medical doctor or pharmacist, signage or showing the availability of FP services, the number of days providing FP services per week, availability of counselling services, types of FP services.

Detailed measures of all these variables are presented in Appendix Table A1.

### Statistical analysis

Since time-invariant variables are dropped in the fixed effects analysis, we used a random effects multivariable Poisson regression model with robust standard error to assess the relationship between the two primary exposures (outlet’s and provider’s characteristics), and the outcome of interest. Marginal effects were computed to examine the direction and magnitude of the relationship. Since each outlet may serve a different population size, a simple regression without weighting may raise a concern of biased estimates. Unfortunately, the CM4FP did not contain weights variable, so we attempted to mitigate the effects of weights by adding: (i) 12 survey sites (four sites for each country); and (ii) population size per 10,000 inhabitants in 2019 or 2020 depending on the availability of statistics for each site^4^. These two additional variables captured potential differences in unobserved factors across sites. Data management and analyses were conducted using Stata SE version 14 (StataCorp, Texas, USA) and a *p*-value of less than 0.1 was considered statistically significant. We used robust standard errors, clustering at the individual level in all estimations.

## Results

### Descriptive results

Descriptive statistics are reported in Table 1. The number of weekly family planning consumers accounted for the highest proportion in Kenya (20.92%), followed by Uganda (12.51%) and Nigeria (7.13%), respectively. The prevalence of non-pharmacy outlets was highest in Uganda and Nigeria (87.20 and 83.92%, respectively), while pharmacy and non-pharmacy rates were approximately equal in Kenya (54.0 and 46.0%, respectively). The percentage of doctors and pharmacists serving in FP service providers in all three countries was very low, with only 5.31% in Kenya, 12.48% in Nigeria, and 26.18% in Uganda. Most outlets have been running for more than 6 months (more than 75%). The percentage of outlets implemented outreach activities was only about 10% across countries. Only a handful of outlets hosted outreach events at community, with the highest percent being Kenya (10%). The corresponding figures for the remaining two countries were below 8%. There were a few outlets showing signs of providing FP services, about 35% in Kenya, 17% in Nigeria, and the lowest was Uganda at 15%. It is interesting to note that only 67.24% of outlets in Nigeria provided counselling service to clients, the corresponding numbers were higher in Uganda and Kenya (76.98 and 88.22%, respectively). The average number of days providing FP services by outlets were quite high, with 6.12, 6.33, and 6.67 days in a week in Kenya, Nigeria and Uganda, respectively. On average, outlets in the three countries provided at least one FP service and two FP services at most.

**Table 1:** Distributions of outlet’s characteristics, provider’s characteristics, and total number of family planning consumers in Kenya, Nigeria and Uganda

	Percent/Mean (SD)			
	Kenya	Nigeria	Uganda	Pooled 3 country
**The number of customers**	20.92 (56.85)	7.13 (11.66)	8.40 (12.51)	10.76 (16.27)
**Outlet’s characteristics**				
Types of outlets				
Not pharmacy	46.19%	83.92%	87.20%	69.82%
Pharmacy	53.81%	16.08%	12.80%	30.18%
Having a medical doctor and/or pharmacist				
No	94.69%	87.52%	73.82%	83.61%
Yes	5.31%	12.48%	26.18%	16.39%
Duration of providing FP services				
Less than 6 months	11.95%	13.14%	24.04%	11.63%
More than 6 months	88.05%	86.86%	75.96%	88.37%
Community outreach activity participation				
No	86.15%	88.07%	90.10%	87.80%
Yes	13.85%	11.93%	9.90%	12.20%
Community outreach event hosting participation				
No	90.35%	11.93%	92.60%	92.02%
Yes	9.65%	6.55%	7.40%	7.98%
Having FP signs				
No	64.85%	82.76%	84.40%	76.19%
Yes	35.15%	17.24%	15.60%	23.81%
Counselling services				
No	11.78%	32.76%	23.02%	22.61%
Yes	88.22%	67.24%	76.98%	77.39%
Days providing FP services	6.12 (1.24)	6.33 (1.34)	6.67 (0.95)	6.32 (1.24)
Number of FP services	2.04 (2.10)	1.25 (1.76)	1.86 (1.89)	1.71 (1.96)
**Provider’s characteristics**				
Sex				
Male	43.34%	39.35%	33.44%	39.51%
Female	56.66%	60.65%	66.56%	60.49%
Age	35.96 (10.74)	39.10 (10.99)	30.69 (8.67)	36.00 (10.88)
Working experience	4.49 (5.28)	8.47 (9.02)	2.83 (4.09)	5.68 (7.20)
Level of education				
Less than University/College	11.57%	26.83%	15.95%	18.58%
University/college	88.43%	73.17%	84.05%	81.42%
FP training received				
No	77.33%	79.44%	73.57%	77.30%
Yes	22.67%	20.56%	26.43%	22.70%

Note: Author’s calculation based on CM4FP Kenya, Nigeria and Uganda longitudinal dataset 2019–2020. Standard deviations are in parentheses. FP means family planning

In terms of provider’s characteristics, it is worth noting that most FP service providers were women, accounting for nearly 60% in all three countries. The average age of service providers was between 30 and 39 years old in the three countries. Service providers in Nigeria had the highest working experience (8.47 years), followed by Kenya and Uganda (4.49 and 2.83 years, respectively). The proportion of service providers with university/college education was quite high, 88.43% in Kenya, 84.05% in Uganda, and 73.17% in Nigeria. However, only about 20% of them received FP training.

### Marginal effects results

**Fig. 1:**
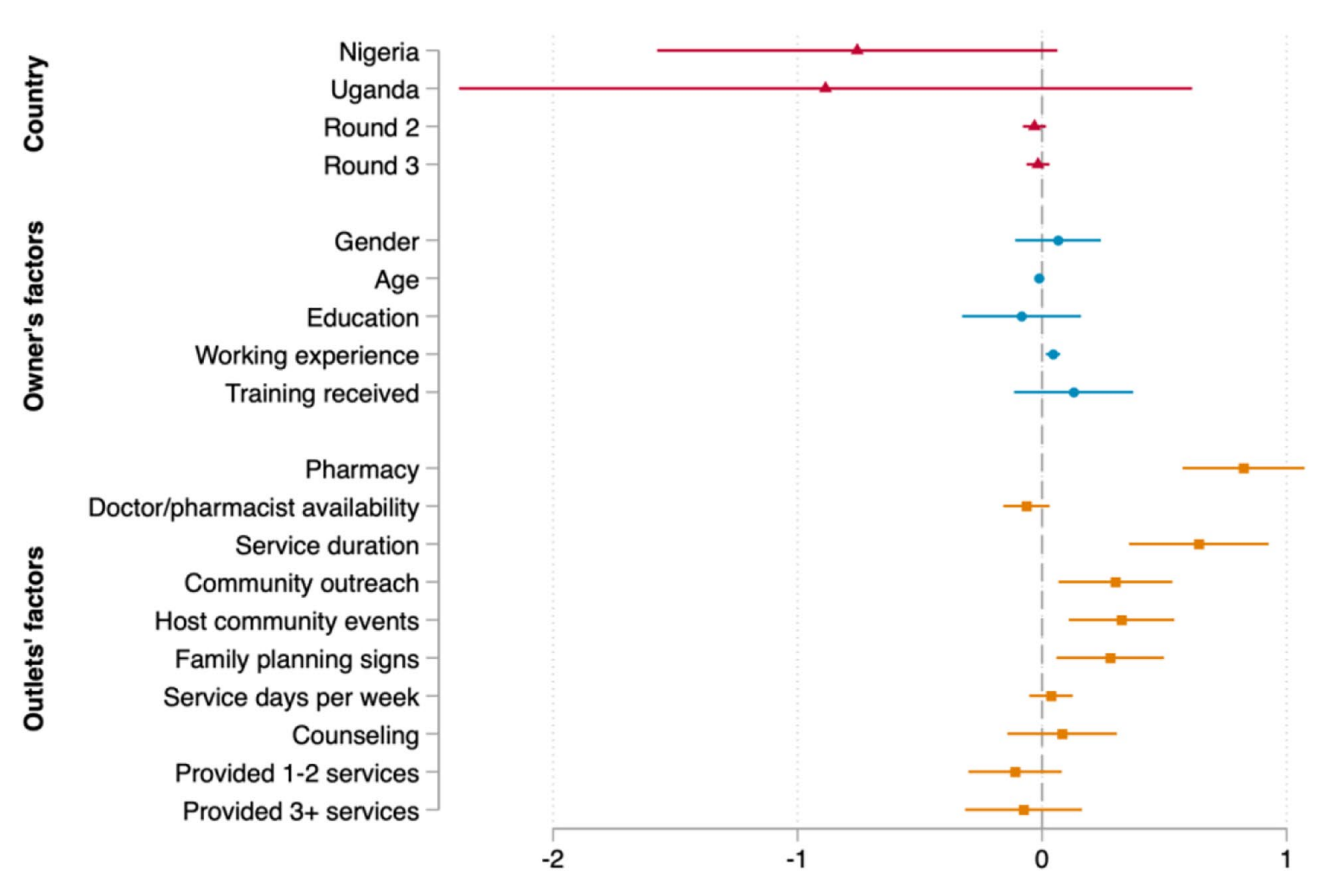
Marginal effects of the association between outlet’s characteristics, provider’s characteristics and family planning consumers for the pooled data

Source: Author’s calculation based on CM4FP dataset.

In this section, we first reported the marginal effects results of the pooled data, followed by the results disaggregated by each country. Specifically, Fig. 1 exhibits the association between the number of customers and provider’s and outlets’ characteristics with 95% confidence interval for the pooled data. In this model, we controlled for country, round dummies and population size per 10,000 inhabitants to account for any potential variations due to changes in economic situations across countries and over time. Regarding the outlets’ characteristics, we found positive associations between the number of customers per week and being a pharmacy, having more days serving per week, participating in community outreach, hosting community events and having FP signs. As for providers’ characteristics, we only found significant effect for working experience, e.g., on average, providers with longer working experience were associated with an increase in the expected number of customers per week in the three countries, holding other variables constant. Precise values of marginal effects of the pooled data were presented in column 5 in Appendix Table A3.

**Fig. 2:**
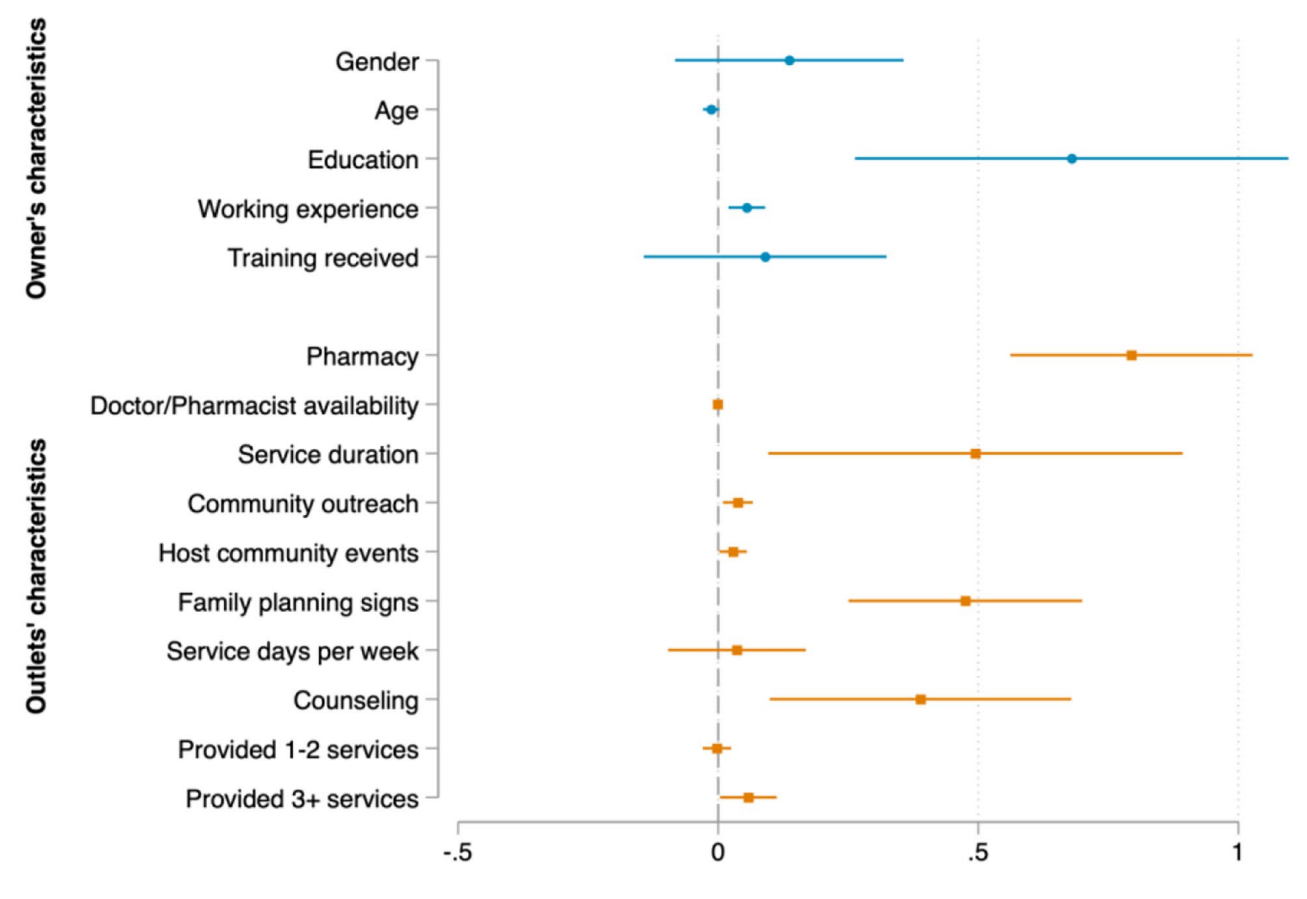
Marginal effects of the association between outlet’s characteristics, provider’s characteristics and Family Planning consumers in Kenya

Source: Author’s calculation based on CM4FP dataset.

**Fig. 3:**
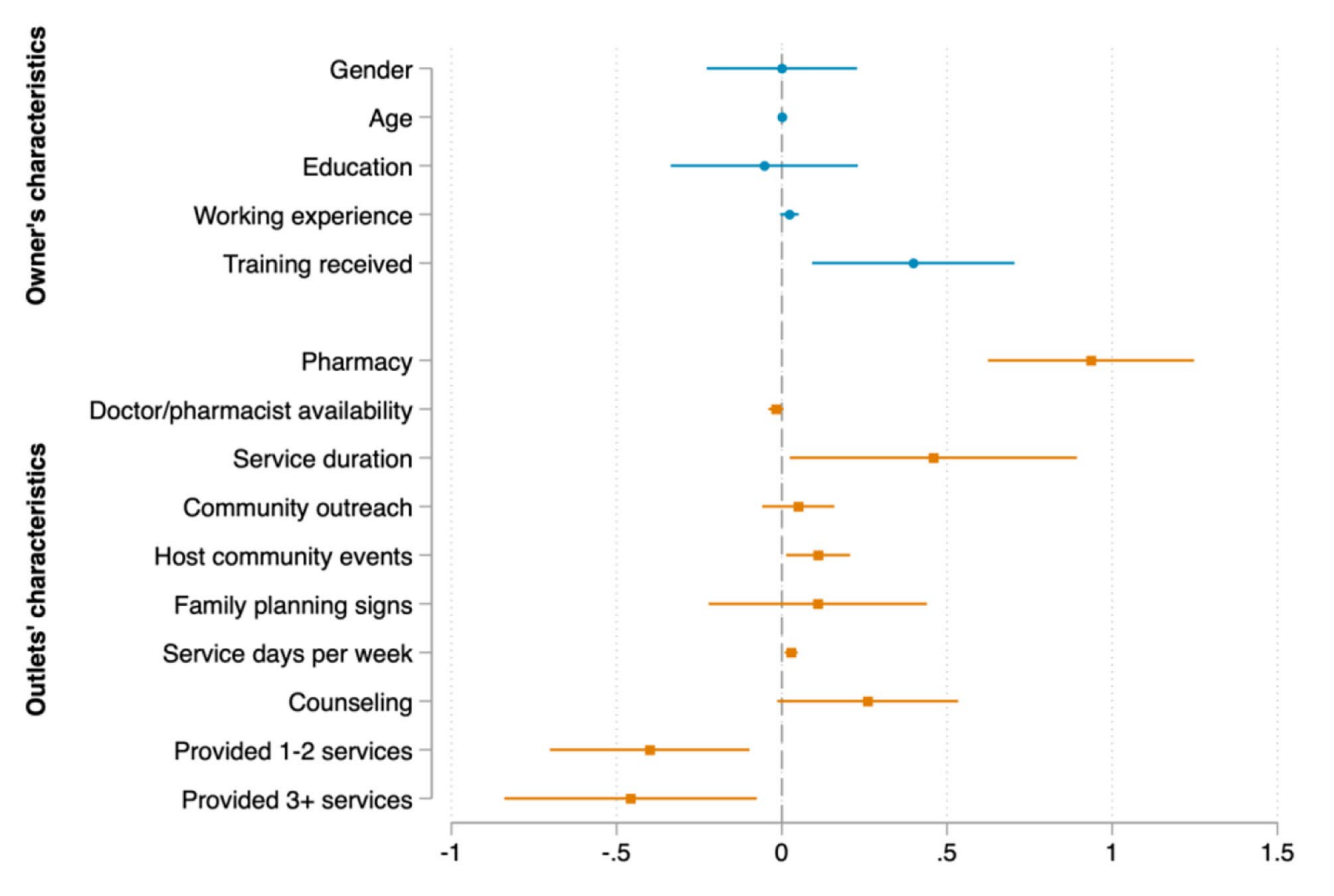
Marginal effects of the association between outlet’s characteristics, provider’s characteristics and family planning consumers in Nigeria

Source: Author’s calculation based on CM4FP dataset.

**Fig. 4:**
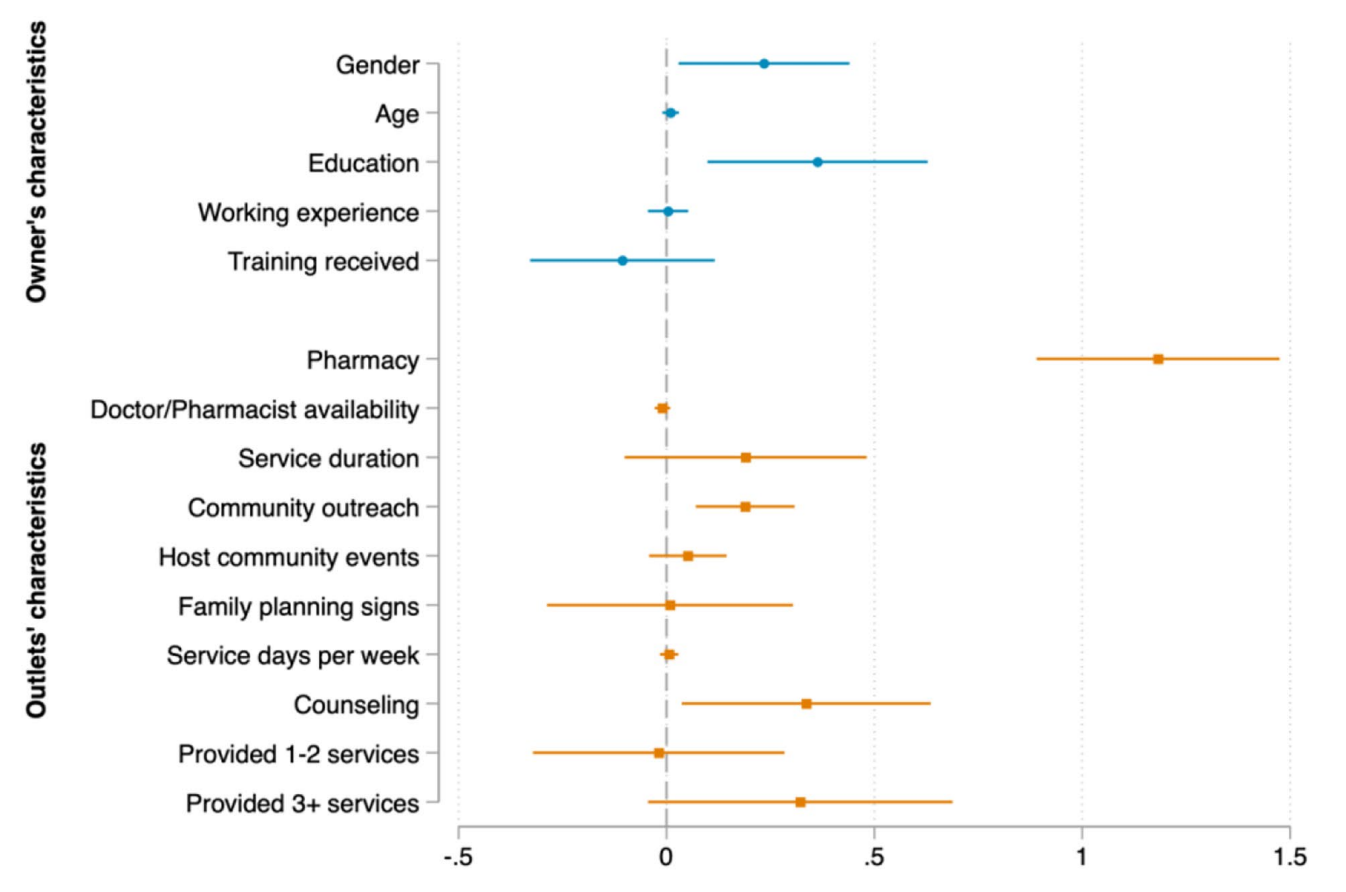
Marginal effects of the association between outlet’s characteristics, provider’s characteristics and family planning consumers in Uganda

Source: Author’s calculation based on CM4FP dataset.

We now turn to the determinants of the number of consumers in each country, which are presented in Figs. 2, 3 and 4. The results reveal that the determinants vary across the countries under examination. As for outlet’s characteristics, given that other predictor variables in the model were held constant, on average, being a pharmacy store was associated with an increase in the expected number of customers per week by 0.760 in Kenya, 0.936 in Nigeria, and 1.182 in Uganda (see Appendix Table A3 for precise values of the marginal effects). Providing FP services in a longer time and hosting family planning outreach events at community were significant determinants in Kenya and Nigeria, but not in Uganda. Participating in community outreach activities was significantly associated with increased the number of consumers in Kenya and Uganda, but not in Nigeria. Having FP signs, providing FP counseling, and providing more FP products (3–5 products, not counting condom) were only found significant in Kenya. Similarly, the number of days serving FP per week was only found significant in Nigeria. Interestingly, we found that providing more FP products other than condom was associated with a decrease in the number of customers in Nigeria.

Regarding providers’ characteristics, longer working experience was only associated with an increase in the average number of PF consumers in Kenya, while receiving FP training within the last 12 months was only found significant in Nigeria. In addition, higher education of providers (college/university and above) was a significant determinant in Kenya and Uganda, but not in Nigeria.

## Discussion

In this study, we utilized a unique panel survey on family planning outlets, a component of the CM4FP, to examine the determinants of the number of FP consumers in Kenya, Nigeria and Uganda, with a focus on outlet’s and provider’s characteristics which are important factors influencing the choice of using contraceptive methods but largely unexplored in previous literature. Our findings show that both outlet’s and provider’s characteristics play important roles in determining the number of customers, and that the determinants vary across the countries under examination.

Regarding outlet’s characteristics, the result of this study underlines the core role of drug stores or pharmacies in being the primary source of modern contraceptives beyond condoms in the three countries under examination. This result is similar to that of Corroon, Kebede [36] about the attraction of private facilities in terms of providing emergency contraceptive pills and oral contraceptives. In addition, it has been reported that pharmacies play an important role in bringing modern contraceptives to consumers in Kenya and Uganda due to its advantages on convenience in time (less waiting time), geographical location and privacy of personal information [7, 37–39]. Therefore, governments could consider improving the availability and appropriate distribution of FP services in private institutions such as pharmacies in order to assure appropriate quantities of each contraceptive technique and to avoid contraceptive shortages in these facilities. In other words, this strategy may guarantee that the needs of using contraceptives among women are properly met in Kenya’s, Nigeria’s and Uganda’s public health facilities.

Our results show that service providers receiving FP training were associated with increased the expected number of customers in Nigeria. Hebert et al. (2013) claimed that many Nigerian family planning practitioners lack formal training, expertise, and abilities in family planning [40]. Therefore, consumers tend to look for service providers with better knowledge and skills about FP. Likewise, the more educated the provider, the more customers it attracts.

Similarly, outlets providing FP consultation tend to increase the number of consumers across the three countries. Tripney, Bird [41] have documented that counseling not only helps attract the number of women seeking FP service, but also increases the effectiveness of contraceptive use and reduces unwanted abortion in low-income countries. In addition, counseling is also considered one of the effective activities to increase the availability of FP services and increase the comfort of customers when using the service [42]. Therefore, in parallel with expanding the distribution of contraceptives to private facilities like pharmacies, FP policy should provide and strengthen training activities to providers to improve professional knowledge on FP in order to gain trust and increase the number of customers seeking FP services. When FP workers at outlets are provided with adequate knowledge, consumers are also expected to receive accurate information and appropriate skills in using FP services through on-site or community-based counseling activities. This practice also increases the acceptance rates to use FP services [43].

The results in Kenya suggest that if the facilities clearly display signs of providing family planning services, there is a tendency to increase the number of consumers. This result is interesting because it runs counter to Abraham, Sick [44]’s research on patient’s decision making in choosing FP providers [44]. Specifically, patients tend to choose providers who continuously meet patients’ needs and have appointment availability instead of advertisements of FP services. In addition, our results also show that longer working experience, longer duration in providing FP services and providing FP service regularly were significantly associated with increased the number of customers. In other words, enhancing the characteristics that are considered professionalism of the service provider is expected to increase the trust of customers [44]. Participating in community outreach activities or hosting community outreach events tends to boost the number of FP consumers who use contraceptives at their stores. This is in line with previous research in Rwanda, a country in Africa with similar socioeconomic characteristics to Kenya, Nigeria and Uganda, showing that social marketing, rather than focusing solely on advertising and sales activities on the premises, plays a significant role in the choice of FP providers [45]. Female providers tend to attract more consumers compared to males in Uganda. This result corresponds to Nalwadda, Mirembe [43]’s study on the obstacles faced by young Ugandans in seeking FP services, provider gender can be considered as a factor causing reluctance to access services [43].

We acknowledge some limitations in our study. First, due to the data structure, we were unable to perform fixed effects regressions that could help eliminate time-invariant heterogenous factors across outlets. Second, the CM4FP is not a national family planning dataset, so the results are not representative for the three countries. Third, although the CM4FP outlets data contains rich information on outlet’ and provider’ characteristics, still it lacks some important information which could be associated with the number of customers such as the size of the outlet, the quality of the outlet, the quality of providers, the distance between outlet and customer’s household, customer service attitude, socioeconomic status of consumers, and so on. Therefore, future research is encouraged to go deeper into characteristics of both outlets and service providers that were not included in this study.

## Conclusion

The findings of this study highlight the crucial role of drug shops or pharmacies as being the main supplier of contemporary contraceptive methods in addition to condoms in the three nations. In general, supply-side initiatives such as providing more service days per week, participating in community outreach, hosting community events, having FP signs, and having providers with more experience in serving FP at the outlets, are probably crucial in drawing customers’ attention to the outlets in all three countries. Additionally, the typical characteristics of providers, such as being female consultant, having deep experience in FP services or having a high level of education and being trained in FP activities, play a significant role in drawing in a sizable clientele. These characteristics are related to professional competence, adequate knowledge, and skills in FP practice from outlets and providers. Therefore, since the percentage of women who use FP is still low in African and other underdeveloped nations, enhancing the qualities of providers and outlets is one of the key strategies for attracting FP customers. According to our research findings, the government’s family planning budgets should instead place an emphasis on small, private businesses like pharmacies or drug stores, where privacy is guaranteed, wait times are minimized, and geographic distance is advantageous when providing sensitive and urgent services like family planning. Additionally, it’s important to focus on several crucial tasks to enhance the qualities of outlets and providers to attract customers, including making sure that they are eye-catching, advertising FP services, have professional credentials, fulfil providers’ obligations to counsel users of contraceptives, provide long-term services and community care, and have female providers.

In summary, the findings of this comparative study might indicate parallels and variances in this connection across three nations under examination. As a result, the study is critical to FP policies and the management of the number of FP consumers in each nation. Furthermore, the discovery of differences in the determinants of outlets and providers on FP consumers in the three countries suggests that more study needs be done to determine the origins of these discrepancies.

### Electronic supplementary material

Below is the link to the electronic supplementary material.

Supplementary Material 1

## Data Availability

Data can be made available from Corresponding Author upon reasonable request. Data from the CM4FP study are available through the Data verse repository, using the following links: Kenya: 10.7910/DVN/DJXKVA. Nigeria 10.7910/DVN/G31FHL. Uganda: 10.7910/DVN/1NRFSD.

## Footnotes

1 Given the objective of our paper, we do not use data from the household surveys, and thus we do not describe its detailed sampling methodologies here. Please refer to Conlon M, Buyungo P, Njogu J, et al.^28^ for details on the household survey.

2 Facilities in this study include health centres/medical centres; hospitals (e.g., general hospital, maternity hospital); pharmacy/chemist, medical clinic/consultancy (e.g., family Planning clinic, maternal child health and family planning clinic, HIV clinic), and dispensary.

3 A person offering family planning services at the time the survey implemented is referred to as a service provider. The study gathers data on the amount of time spent on providing FP services for both the outlet and provider.

4 Data on population size was taken from https://www.citypopulation.de.
