# Supplementary material for: What do outlet’s and provider’s characteristics mean for family planning consumers? A comparative study of Kenya, Nigeria and Uganda
Source: BMC Womens Health. 2023 Oct 16;23:537. doi: 10.1186/s12905-023-02699-0 (PMC10580626; doi:10.1186/s12905-023-02699-0)
Supplement: Supplementary file 1 — Supplementary Material 1 [file 12905_2023_2699_MOESM1_ESM.docx]

**Appendix Tables**

**Table A1**: *Variable measurements*

| ***Variable*** | ***Measurement*** |
| --- | --- |
| **Average total number of FP consumers^1^** | - Pooled data (min = 0, max = 100)  - Kenya (min = 0, max = 100)  - Nigeria (min = 0, max = 100)  - Uganda (min = 0, max = 100) |
| **Outlet’s characteristics** |  |
| Type of outlet^2^ | 0 = not pharmacy; 1 = pharmacy |
| Having a medical doctor and/or pharmacist | 0 = no; 1 = yes |
| Duration of providing FP services | 0 = less than 6 months; 1 = more than 6 months |
| Community outreach activity participation | 0 = no; 1 = yes |
| Outreach event hosting participation | 0 = no; 1 = yes |
| Days providing FP services | - Pooled data (min = 1, max = 7)  - Kenya (min = 1, max = 7)  - Nigeria (min = 1, max = 7)  - Uganda (min = 1, max = 7) |
| Having FP signs | 0 = no; 1 = yes |
| Counselling services | 0 = no; 1 = yes |
| Number of FP services | 0 = only condom, 1 = 1-2 products, 2 = 3-5 products |
| **Provider’s characteristics** |  |
| Sex | 0 = male, 1 = female |
| Age | - Pooled data (min = 18, max = 75)  - Kenya (min = 19, max = 74)  - Nigeria (min = 18, max = 75)  - Uganda (min = 19, max = 70) |
| Working experience | - Pooled data (min = 0, max = 49)  - Kenya (min = 0, max = 41)  - Nigeria (min = 0, max = 49)  - Uganda (min = 1, max = 25) |
| FP training received | 0 = no; 1 = yes |
| Level of education | 0 = less than university/college; 1 = university/college |

Note: FP means family planning

^1^We top-coded the number of customers at 100

^2^ The dataset contains a variety of different types of outlets, such as health centres, hospital, pharmacy, medical clinic, and others. However, we separated pharmacy and non-pharmacy because the percentage distribution for each type of outlet was rather small. Non-pharmacy includes health centres/medical centres, hospital, medical clinic/consultancy (e.g., family planning clinic, maternal child Health and family planning clinic, HIV clinic), and dispensary.

**Table A2:** *Percentage distribution of outlets that serve modern contraceptive methods by countries*

|  | **Kenya** | **Nigeria** | **Uganda** |
| --- | --- | --- | --- |
| Oral contraceptive tablets |  |  |  |
| No | 18.38 | 47.99 | 50.81 |
| Yes | 81.62 | 52.01 | 49.19 |
| Emergency contraceptive tablets |  |  |  |
| No | 33.68 | 62.54 | 47.17 |
| Yes | 66.32 | 37.46 | 52.83 |
| Injectable contraceptives |  |  |  |
| No | 62.37 | 70.40 | 52.43 |
| Yes | 37.63 | 29.60 | 47.57 |
| Contraceptive implants |  |  |  |
| No | 74.91 | 87.79 | 82.18 |
| Yes | 25.09 | 12.21 | 17.82 |
| Contraceptive IUDs |  |  |  |
| No | 81.10 | 89.63 | 86.03 |
| Yes | 18.90 | 10.37 | 13.97 |
| Oral contraceptive tablets |  |  |  |
| No | 41.67 | 66.06 | 43.90 |
| Yes | 58.33 | 33.94 | 56.10 |
| Emergency contraceptive tablets |  |  |  |
| No | 79.17 | 80.49 | 48.78 |
| Yes | 20.83 | 19.51 | 51.22 |
| Contraceptive implants |  |  |  |
| No | 97.92 | 98.17 | 95.12 |
| Yes | 2.08 | 1.83 | 4.88 |
| Contraceptive IUDs |  |  |  |
| No | 100.00 | 97.56 | 98.78 |
| Yes | 0.00 | 2.44 | 1.22 |

**Table A3:** *Marginal effects of the link between outlet’s characteristics, provider’s characteristics and family planning consumers*

|  | **Kenya** | **Nigeria** | **Uganda** | **Pooled 3 country** |
| --- | --- | --- | --- | --- |
| **Country** (Ref. Kenya) |  |  |  |  |
| Nigeria | - | - | - | -0.756 (0.418) |
| Uganda | - | - | - | -0.886 (0.765) |
| **Outlet’s characteristics** |  |  |  |  |
| Type of outlet (Ref. not pharmacy) |  |  |  |  |
| Pharmacy | 0.760^***^ (0.121) | 0.936^***^ (0.159) | 1.182^***^ (0.149) | 0.824^***^ (0.127) |
| Having a medical doctor and/or pharmacist (Ref. no) | -0.002 (0.003) | -0.018 (0.012) | -0.010 (0.010) | -0.064 (0.048) |
| Duration of providing FP services (Ref. <6 months) |  |  |  |  |
| > 6 months | 0.481^**^ (0.201) | 0.459^**^ (0.222) | 0.190 (0.149) | 0.641^***^ (0.146) |
| Community outreach activity participation (Ref. no) | 0.038^***^ (0.015) | 0.050 (0.056) | 0.189^***^ (0.061) | 0.300^**^ (0.119) |
| Outreach event hosting participation (Ref. no) | 0.032^**^ (0.013) | 0.110^**^ (0.049) | 0.051 (0.048) | 0.325^***^ (0.110) |
| Having FP signs (Ref. no) | 0.472^***^ (0.115) | 0.109 (0.169) | 0.008 (0.151) | 0.278^**^ (0.112) |
| Days providing FP services | 0.023 (0.068) | 0.028^***^ (0.010) | 0.006 (0.011) | 0.036 (0.045) |
| Counselling services (Ref. no) | 0.398^***^ (0.148) | 0.260^*^ (0.140) | 0.336^**^ (0.153) | 0.082 (0.114) |
| Number of FP services (Ref. only condom) |  |  |  |  |
| 1-2 products | -0.001 (0.014) | -0.400^***^ (0.154) | -0.019 (0.154) | -0.111 (0.097) |
| 3-5 products | 0.061^**^ (0.028) | -0.459^**^ (0.194) | 0.322^*^ (0.187) | -0.076 (0.112) |
| **Provider’s characteristics** |  |  |  |  |
| Sex (Ref. male) | 0.124 (0.111) | 0.001 (0.116) | 0.234^**^ (0.105) | 0.065 (0.089) |
| Age | -0.013^*^ (0.008) | 0.001 (0.007) | 0.010 (0.010) | -0.013^*^ (0.007) |
| Working experiences | 0.053^***^ (0.018) | 0.023 (0.014) | 0.004 (0.025) | 0.045^***^ (0.015) |
| FP training received (Ref. no) | 0.088 (0.118) | 0.398^**^ (0.156) | -0.106 (0.113) | 0.129 (0.124) |
| Level of education (Ref. less than university/college) |  |  |  |  |
| University/college and above | 0.597^***^ (0.223) | -0.053 (0.145) | 0.363^**^ (0.135) | -0.084 (0.124) |
| County | -0.209 (0.118) | -0.004 (0.006) | 0.347^***^ (0.125) | 0.057 (0.087) |
| Population size per 10,000 inhabitants | -0.001 (0.001) | -0.001 (0.002) | 0.001^**^ (0.000) | -0.001 (0.001) |
| **Total number of observations** | **1,321** | **1,255** | **842** | **3,418** |

Source: Author’s calculation based on CM4FP Kenya, Nigeria and Uganda longitudinal dataset 2019-2020

*** p<0.01; ** p<0.05; * p<0.1. Marginal effects are reported for Poisson regression models (random effects). Standard errors are in the parentheses. Ref. denotes reference group. FP stands for family planning.
